# Risk Factors and Outcomes of Acute Graft Pyelonephritis with Bacteremia Due to Multidrug-Resistant Gram-Negative Bacilli among Kidney Transplant Recipients

**DOI:** 10.3390/jcm11113165

**Published:** 2022-06-02

**Authors:** Núria Sabé, Marta Maristany, Manel Tuells, Alexandre Favà, Edoardo Melilli, Fe Tubau, Josep Maria Cruzado, Jordi Carratalà

**Affiliations:** 1 Infectious Disease Department, Bellvitge University Hospital-IDIBELL, University of Barcelona, 08907 Barcelona, Spain; mmaristany@bellvitgehospital.cat (M.M.); mtuells@bellvitgehospital.cat (M.T.); 2 Centro de Investigación Biomédica en Red de Enfermedades Infecciosas (CIBERINFEC), Instituto de Salud Carlos III, 28029 Madrid, Spain; 3 Kidney Transplant Unit, Nephrology Department, Bellvitge University Hospital-IDIBELL, University of Barcelona, 08907 Barcelona, Spain; afava@bellvitgehospital.cat (A.F.); emelilli@bellvitgehospital.cat (E.M.); jmcruzado@bellvitgehospital.cat (J.M.C.); 4 Spanish Network for Renal Research (REDINREN), Instituto de Salud Carlos III, 28029 Madrid, Spain; 5 Microbiology Department, Bellvitge University Hospital-IDIBELL, 08907 Barcelona, Spain; f.tubau@bellvitgehospital.cat; 6 Centro de Investigación Biomédica en Red de Enfermedades Respiratorias (CIBERES), Instituto de Salud Carlos III, 28029 Madrid, Spain

**Keywords:** kidney transplant, acute graft pyelonephritis, bacteremia, bloodstream infection, multidrug-resistant

## Abstract

Acute graft pyelonephritis (AGP) is the leading cause of bloodstream infection in kidney transplant (KT) recipients. The prevalence of urinary tract infections caused by multidrug-resistant (MDR) Gram-negative bacilli is increasing. This 14-year prospective observational study sought to determine the clinical characteristics, risk factors, and outcomes of AGP with bacteremia due to MDR Gram-negative bacilli. Overall, 278 episodes of AGP with bacteremia due to MDR Gram-negative and non-MDR Gram-negative bacilli were identified and compared in 214 KT recipients; MDR Gram-negative bacilli were the cause in 28.4%. Overall 30-day mortality was low (1.1%). Risk factors independently associated with AGP due to MDR Gram-negative bacilli were male sex (OR 3.08; 95%CI 1.60–5.93), previous episode of bacteremic AGP (OR 2.11, 95%CI 1.09–4.09), prior antibiotic therapy in the preceding month (OR 2.47, 95%CI 1.33–4.57), and nosocomial acquisition (OR 2.03, 95%CI 1.14–3.62). Forty-three percent of MDR Gram-negative episodes received inappropriate empirical antibiotic therapy. The risk factors identified in this study may help physicians when selecting empirical antibiotic treatment for AGP. Previous antibiotic use was the main modifiable factor. Its presence highlights the importance of avoiding unnecessary antibiotics in order to bring down the high rates of MDR Gram-negative bacilli infections in this population.

## 1. Introduction

Today, bacterial infections have overtaken classical opportunistic infections as the most frequent complication among solid organ transplant (SOT) recipients [1]. In this population, bloodstream infection (BSI) is one of the most challenging clinical issues. BSIs occur in over one-third of SOT recipients, and have classically been associated with high mortality rates [2,3]. Urinary tract infections (UTIs) are the most common infectious complication in kidney transplant (KT) recipients, especially in the first months after transplantation [4,5,6]. Acute graft pyelonephritis (AGP) is the most severe manifestation of UTI in this population, with an estimated rate between 10 and 20% [4,7,8,9], and is the leading cause of BSI in KT recipients [4,5,9,10]. Moreover, AGP has been associated with kidney graft function impairment, graft loss, and mortality [9,11,12,13]. New measures are needed to improve the management of AGP after KT. Importantly, the potential preventive role of antibiotic treatment for asymptomatic bacteriuria has been questioned in randomized trials and, in fact, there is little evidence to support its use [14].

Currently, the emergence of multidrug-resistant (MDR) organisms is causing worldwide concern. In KT recipients, rates of UTI due to MDR Gram-negative bacilli have increased alarmingly in recent years [15,16]. MDR Gram-negative bacilli infections in this population have been associated with a higher incidence of complications, mortality, and recurrence [3,4,17]. The limited development of new antibiotics has meant that MDR infections are difficult to treat, and the treatment options available may involve the use of agents with elevated renal toxicity. Identifying risk factors associated with AGP with bacteremia due to MDR Gram-negative bacilli is of paramount importance in order to be able to implement appropriate preventive and therapeutic strategies. This study aimed to determine the clinical characteristics, risk factors, and outcomes of MDR Gram-negative bacilli bacteremic AGP in KT recipients.

## 2. Materials and Methods

### 2.1. Setting and Study Population

We conducted a prospective observational study at a tertiary university referral hospital in Barcelona, Spain, with an active kidney transplantation program. From 1 February 2007 to 30 November 2021, all consecutive episodes of BSI occurring in hospitalized adult KT recipients were included. Data regarding baseline characteristics, as well as immunosuppressive treatment, occurrence of acute allograft rejection and opportunistic infections, clinical features, microbiological studies, and outcomes, were carefully recorded in a specific database. For the purposes of this study, cases of AGP with bacteremia in KT recipients were selected for retrospective analysis. We compared clinical characteristics and outcomes of MDR Gram-negative and non-MDR Gram-negative bacteremic AGP to identify the risk factors for the form of the disease caused by MDR Gram-negative bacilli in KT recipients. The present study was approved by our institution’s ethics committee.

### 2.2. Microbiological Studies

Blood samples were processed via the BACTEC 9240 method (Becton-Dickinson Microbiology Systems, Sparks, MD, USA). The inoculated bottles were incubated for 5 days at 35 °C before being discharged. Microbial identification was performed with commercially available panels—MicroScan (Siemens Healthcare Diagnosis Inc; West Sacramento, CA, USA) or VITEK (bioMérieux SA; Marcy-L’Etoile, France)—with standard biochemical and/or enzymatic tests, or with a matrix-assisted laser desorption/ionization time-of-flight mass spectrometry (MALDI-TOF) Biotyper^®^ system (Bruker, Billerica, MA, USA). Antibiotic susceptibility was determined by disk-diffusion and microdilution methods, using the MicroScan^®^ automated system (Beckman Coulter Inc., Brea, CA, USA) or VITEK (bioMérieux SA; Marcy-L’Etoile, France). All susceptibility studies and phenotype characterization of ESBL and/or carbapenemase production were performed and interpreted according to EUCAST guidelines (http://www.eucast.org/clinical_breakpoints/, accessed on 1 January 2022). At our hospital, the microbiology laboratory reports all positive results of blood cultures to the infectious disease team daily. All KT recipients with BSI are visited by an infectious disease physician and followed-up. Changes in antimicrobial treatment and general management are discussed with attending transplant physicians.

### 2.3. Clinical Data, Definitions, and Prophylaxis

Bacteremic AGP was defined as the presence of bacteremia with clinical symptoms of renal allograft tenderness, chills, or dysuria or urinary urgency, and isolation of an organism from a urine culture matched with that obtained in blood cultures based on species identification and antibiotic susceptibility results. An episode was considered as new if the patient had been asymptomatic for a period of at least one month and had then become reinfected. A prior episode of AGP with bacteremia was considered when another episode was documented during the study period. In accordance with the current standard definition, multidrug resistance was defined as acquired non-susceptibility to at least one agent in three or more antimicrobial categories [18]. Renal impairment was defined as a serum creatinine level of > 1.5 mg/dL. Prior antibiotic therapy was defined as the receipt of any systemic antibiotic for 48 h or more in the previous month. Empirical antibiotic therapy was considered inappropriate if the treatment regimen did not include at least one antibiotic that was active in vitro against the infecting microorganism. Septic shock was defined as sepsis-induced hypotension persisting despite adequate fluid resuscitation, as described elsewhere [19]. Acute allograft rejection was considered when demonstrated by biopsy. Opportunistic infection was considered when any infection due to an opportunistic pathogen occurred during the bacteremic AGP episode. Overall mortality was defined as death by any cause within the first 30 days of the onset of bacteremic AGP.

KT recipients received perioperative antibacterial prophylaxis with intravenous amoxicillin clavulanate or cefuroxime. Prophylaxis to prevent *Pneumocystis jirovecii* infection with trimethoprim–sulfamethoxazole (a double-strength tablet taken once three times a week) was given in the first 3 to 6 months after transplantation. A prophylactic approach was also used for recipients at high risk of cytomegalovirus disease (e.g., D+/R– recipients, and recipients who received anti-thymocyte globulin). In the other cases, pre-emptive therapy was administered.

### 2.4. Statistical Analysis

In the comparative analysis, we used the chi-squared test with continuity correction for categorical variables. Continuous data are presented as the median (interquartile range (IQR)), and were analyzed with the Mann–Whitney U test. Statistically significant variables in the univariate analysis and clinically relevant variables were entered into a multivariate conditional model for logistic regression analysis, and the OR and 95% confidence interval (CI) were calculated. Age and gender were included in the final regression model for theoretical reasons. The analysis was performed with the stepwise logistic regression model of SPSS version 18.0 (SPSS, Chicago, IL, USA). All statistical tests were two-tailed, and the threshold of statistical significance was set at *p* < 0.05.

## 3. Results

### 3.1. Demographic Characteristics, Clinical Characteristics, and Mortality

During the study period, 278 episodes of AGP with bacteremia were identified in 214 KT recipients. The demographic characteristics, clinical characteristics, and mortality associated with these episodes are detailed in Table 1. One KT recipient had concomitant liver transplantation. Fifty percent of patients had received antibiotic therapy in the previous month, and twenty-three percent presented a previous episode of bacteremic AGP. Nearly half of the episodes were nosocomially acquired.

Sixty-eight (24.5%) patients received inappropriate empirical antibiotic treatment, but the overall 30-day mortality of bacteremic AGP in KT recipients was low (1.1%). The three patients who died presented septic shock, and two of them required mechanical ventilation and intensive care unit admission. Multi-organ failure was the cause of death in all cases. One of these episodes was due to ESBL-producing *Klebsiella pneumoniae*, one due to *Enterococcus faecalis*, and the other due to *Enterococcus faecium*.

### 3.2. Causative Microorganisms

The microbiological etiology of the 278 episodes of bacteremic AGP is detailed in Table 2. *Escherichia coli* was the most frequent causative microorganism, followed by *Klebsiella* spp. and *Pseudomonas* spp. Among the 225 Enterobacteriaceae isolates, 27.5% were ESBL-producing and 2.2% were carbapenemase-producing. About half of the *Pseudomonas* spp. strains were MDR. Overall, MDR Gram-negative bacilli caused 79 (28.4%) of the episodes of AGP with bacteremia.

### 3.3. Comparison of Demographic and Clinical Characteristics of MDR and Non-MDR Gram-Negative Bacilli AGP with Bacteremia

No differences were found when comparing the baseline characteristics of bacteremic episodes of AGP caused by MDR and by non-MDR Gram-negative bacilli regarding immunosuppressive treatment or previous allograft rejection (Table 3). Underlying diseases and clinical and laboratory data at admission were also comparable in both groups. However, AGP with bacteremia due to MDR Gram-negative bacilli was more frequent in older males, and occurred mainly in the first year after transplantation, in a nosocomial setting. Post-transplant surgical complications, prior antibiotic treatment in the last month, and prior episodes of AGP were also more common in cases caused by MDR Gram-negative bacilli. As MDR Gram-negative bacilli AGP with bacteremia was more frequent in male KT recipients than in females, a sub-analysis was performed comparing baseline characteristics and causative organisms of the bacteremic AGP episodes according to the patient’s gender. The median age of KT recipients with bacteremic AGP was higher in males than in females, but without statistical significance (67 vs. 65 years; *p* = 0.065). Male patients with bacteremic AGP were more likely to have a urethral catheter at the time of BSI (27.3% vs. 18.9%; *p* = 0.003). Additionally, episodes of AGP in men were more often nosocomially acquired (49.7% vs. 35.5%; *p* = 0.020) (Supplementary Table S1). Regarding causative organisms of AGP with bacteremia, *Escherichia coli* was less frequent in male KT recipients than in females (34.5% vs. 66.4%; *p* < 0.001). Male KT recipients with bacteremic AGP also were more likely to have *Pseudomonas* spp. (18.7% vs. 2.8% *p* < 0.001) and *Enterococcus* spp. (9.4% vs. 1.9%; *p* = 0.014) isolates (Supplementary Table S2).

### 3.4. Risk Factors for Bacteremic AGP Due to MDR Gram-Negative Bacilli

In the conditional multivariate analysis, male sex (OR 3.08; 95% CI 1.60–5.93), previous episode of bacteremic AGP (OR 2.11, 95% CI 1.09–4.09), prior antibiotic therapy in the preceding month (OR 2.47, 95% CI 1.33–4.57), and nosocomial acquisition (OR 2.03, 95% CI 1.14–3.62) were identified as independent risk factors for MDR Gram-negative AGP with bacteremia (Table 4).

### 3.5. Comparison of Outcomes of MDR and Non-MDR Gram-Negative Bacilli AGP with Bacteremia

Outcomes of episodes of AGP with bacteremia are shown in Table 5. KT recipients with MDR Gram-negative bacilli bacteremic AGP more frequently received inappropriate empirical antibiotic treatment, and needed longer intravenous antibiotic treatment and hospitalization. Opportunistic infections—especially cytomegalovirus infection—were more frequent in AGP with bacteremia due to MDR Gram-negative bacilli. However, other outcomes—such as impairment of renal function, mechanical ventilation, intensive care unit admission, and 30-day mortality—did not show differences between groups.

## 4. Discussion

In this prospective observational study, MDR Gram-negative bacilli caused 28.4% of cases of AGP with bacteremia in KT recipients. Male sex, nosocomial acquisition, prior antibiotic therapy, and previous episodes of bacteremic AGP were independently associated with AGP with bacteremia due to MDR Gram-negative bacilli. Even though inappropriate empirical antibiotic treatment was frequent among MDR Gram-negative episodes, the overall 30-day mortality was very low.

The finding that nearly one-third of the episodes of bacteremic AGP in KT recipients were due to MDR Gram-negative bacilli is a cause for concern. The incidence of UTIs due to MDR Gram-negative bacilli in KT recipients is increasing at an alarming pace worldwide, ranging from 1.3 to 5.8 in different reports [5,15,20,21]. A better understanding of the risk factors for MDR Gram-negative infections in KT recipients is essential in order to identify measures for reducing MDR Gram-negative bacilli infections and for targeting empirical antibiotic therapy.

We found that male gender was independently associated with AGP with bacteremia due to MDR Gram-negative bacilli in KT recipients. Conversely, UTIs were more common in women, but mainly due to a higher incidence of cystitis with benign outcomes. Episodes of bacteremic AGP in our male patients were more frequently associated with the use of urinary catheters and nosocomial acquisition—factors that have previously been associated with UTIs caused by MDR microorganisms in the general population [22]. Moreover, male KT recipients with bacteremic AGP tend to be older, and predisposing factors for UTIs in men—such as prostatic hyperplasia—may be more common [23,24]. All of these factors may help to explain the identification of male sex as a risk factor for MDR Gram-negative episodes in this study.

Nosocomially acquired infection was identified as an independent risk factor for AGP with bacteremia due to MDR Gram-negative bacilli. The high incidence of MDR Gram-negative bacilli in nosocomial UTIs in the general population is well known [22]. In KT recipients, a higher incidence of UTIs due to MDR Gram-negative bacilli has been reported in nosocomial settings [20,25,26]. This finding emphasizes the importance of applying high-quality infection control programs, including reducing the duration of procedures and the use of invasive instrumentation, and introducing improvements in infection control practices [3].

Previous antibiotic use has been identified as a significant risk factor for MDR infections in SOT recipients [2,6,20,27]. In our study, prior antibiotic therapy was also independently associated with AGP with bacteremia due to MDR Gram-negative bacilli, and was the main modifiable risk factor. Rationalizing the use of antibiotics among this population is extremely important in order to decrease rates of antimicrobial resistance [2,3,6]. In contrast, trimethoprim–sulfamethoxazole prophylaxis in our KT recipients with bacteremic AGP was not associated with multidrug resistance, consistent with the results of a previous study in a cohort of SOT recipients with BSIs caused by ESBL-producing Enterobacteriaceae [28]. Previous episodes of bacteremic AGP were also independently associated with MDR Gram-negative etiology. Consistent with this finding, other researchers have associated the etiology of MDR Gram-negative bacilli with recurrent UTIs in KT recipients [17,29].

The 30-day mortality of AGP with bacteremia was low in our study. Although BSIs have been associated with poor outcomes in SOT recipients [30], other studies have reported lower mortality related to BSIs in SOT recipients than in the general population [31]. Moreover, BSIs of urinary origins have lower mortality than other sources of bacteremia [20,32]. BSIs caused by MDR Gram-negative bacilli in SOT recipients have been associated with higher mortality rates, especially in cases of delayed appropriate antibiotic treatment [33]. At our hospital, as soon as the results of blood cultures are obtained, the microbiologists provide the infectious disease team with all of the data regarding the identification of microorganisms and their antibiotic susceptibility. This practice allows the early modification of antibiotic treatments, and may improve the poor prognosis associated with inappropriate empirical antibiotic treatment in MDR Gram-negative BSIs in our KT population. In addition, new antibiotics with greater activity and a better toxicity profile against MDR Gram-negative bacilli are now available, such as ceftolozane–tazobactam, cefiderocol [34,35], or beta-lactam combinations with new beta-lactamase inhibitors [36]—such as ceftazidime–avibactam—that could improve the evolution of infections caused by MDR Gram-negative microorganisms in KT recipients.

Nevertheless, our KT recipients with bacteremic AGP due to MDR Gram-negative bacilli had longer intravenous antibiotic treatment and hospitalization—a circumstance that increased economic costs. Opportunistic infections—especially those caused by cytomegalovirus—were also more common among KT recipients with AGP caused by MDR Gram-negative bacilli, as previously reported elsewhere [27], reflecting the compromised immune response of transplant patients in this situation.

The strengths of this study are the prospective design spanning a 14-year period and the large number of cases. Importantly, although previous studies have evaluated BSIs caused by MDR Gram-negative bacilli in KT recipients, none focused on AGP. However, our study also has some limitations that should be acknowledged: Firstly, UTIs have been associated with poorer graft survival and worse long-term renal function; as our patients were followed up at 30 days after bacteremic AGP, long-term effects could not be assessed. Secondly, our study was conducted in a single center; therefore, the extrapolation of our findings to other settings should be performed with caution.

## 5. Conclusions

KT recipients with AGP with bacteremia presented low 30-day mortality. MDR Gram-negative bacilli caused nearly one-third of the bacteremic AGP episodes. Risk factors associated with AGP with bacteremia due to MDR Gram-negative bacilli were male sex, nosocomial acquisition, prior antibiotic treatment, and previous episodes of bacteremic AGP. The identification of these risk factors may help physicians to reduce the high percentage of inappropriate empirical antibiotic treatment in MDR Gram-negative bacilli AGP. Moreover, previous antibiotic use was the main modifiable factor identified; its high presence highlights the importance of avoiding unnecessary antibiotics in KT recipients in order to bring down the high rates of MDR Gram-negative infections. However, these findings need to be corroborated in future research.

## Figures and Tables

**Table 1:** Demographic characteristics, clinical characteristics, and mortality of 278 episodes of acute graft pyelonephritis with bacteremia in 214 KT recipients.

Variables	*n* = 278 *n* (%)
Male sex	171 (61.5)
Age, years, median (IQR)	66 (58–71)
**Underlying diseases**	258 (92.8)
Arterial hypertension	201 (72.3)
Dyslipidemia	85 (30.6)
Diabetes mellitus	95 (34.3)
Chronic heart disease	88 (31.7)
Chronic pulmonary disease	43 (15.5)
Chronic liver disease	13 (4.7)
Hematological malignancies	4 (1.4)
Solid tumor	17 (6.1)
Obesity	12 (4.3)
Other underlying disease	38 (13.7)
**Etiology of chronic renal insufficiency before transplant**	
Unknown	87 (31.3)
Glomerulonephritis	45 (16.2)
Diabetic nephropathy	43 (15.5)
Kidney polycystic disease	37 (13.3)
Nephroangiosclerosis	27 (9.7)
Other etiologies of chronic renal insufficiency	39 (14.0)
**Living donor**	25 (9.0)
**Previous kidney transplant**	46 (16.5)
**Antimicrobial prophylaxis**	
TMP-SMZ prophylaxis	82 (29.6)
Valganciclovir prophylaxis	36 (13.0)
**Immunosuppressive therapy**	
Prednisone	238 (85.6)
Calcineurin inhibitors	244 (87.8)
mTOR inhibitors	35 (12.6)
Mycophenolate mofetil	248 (89.2)
Lymphocyte-depleting antibody (≤6 months)	88 (31.8)
Anti-thymocyte globulin (≤6 months)	43 (15.6)
≥1 pulse of 1 g of intravenous methylprednisolone (≤6 months)	36 (13.1)
**Acute allograft rejection** (≤6 months)	18 (6.5)
**Prior antibiotic therapy ^1^**	140 (50.4)
**Prior bacteremic acute graft pyelonephritis**	64 (23%)
**Clinical and laboratory findings at presentation**	
Median days from kidney transplantation (IQR)	221 (39–1353)
Nosocomial acquisition	123 (44.2)
Use of urinary catheter	81 (29.2)
Temperature ≥ 38 °C	132 (49.8)
White blood cell count > 10,000/mm^3^	159 (57.2)
Renal impairment at presentation ^2^	78.8 (78.8)
Shock at presentation	13 (4.7)
**Inappropriate empirical antibiotic treatment**	68 (24.5)
**30-day mortality**	3 (1.1)

^1^ Prior antibiotic therapy was defined as the receipt of any systemic antibiotic in the preceding month for 48 h or more. ^2^ Renal impairment was defined as a serum creatinine level > 1.5 mg/dL. Abbreviations: IQR, interquartile range; mTOR, mammalian target of rapamycin; TMP–SMZ, trimethoprim–sulfamethoxazole.

**Table 2:** Causative microorganisms of 278 episodes of acute graft pyelonephritis with bacteremia in 214 KT recipients.

Microorganisms	*n* (%)
**Gram-negative**	
*Escherichia coli*	130 (46.8)
ESBL-producing *Escherichia coli*	20 (7.2)
*Klebsiella* spp.	72 (25.9)
ESBL-producing *Klebsiella* spp.	41 (14.8)
Carbapenemase-producing *Klebsiella* spp.	4 (1.4)
*Pseudomonas* spp.	35 (12.6)
*MDR Pseudomonas*	17 (6.1)
*Enterobacter* spp.	9 (3.2)
*Proteus mirabilis*	7 (2.5)
Other Gram-negative bacilli ^1^	7 (2.5)
*ESBL*-producing Enterobacteriaceae	62 (22.3)
Carbapenemase-producing Enterobacteriaceae	5 (1.8)
MDR Gram-negative bacilli ^2^	79 (28.4)
**Gram-positive**	
*Enterococcus* spp.	18 (6.4)
*Enterococcus faecium*	4 (1.4)
*Staphylococcus aureus*	3 (1.1)
*Lactobacillus* spp.	1 (0.4)
***Candida albicans***	1 (0.4)
**Polymicrobial**	6 (2.2)

^1^ Other Gram-negative bacilli included *Serratia marcescens*, *n* = 3; *Citrobacter freundii*, *n* = 2; *Morganella morganii*, *n* = 1; and *Acinetobacter baumannii*, *n* = 1. ^2^ Multidrug-resistant Gram-negative bacilli included ESBL-producing *Enterobacteriaceae, n =* 60; carbapenemase-producing Enterobacteriaceae, *n* = 5; MDR *Pseudomonas* spp., *n* = 17; and *Acinetobacter baumannii*, *n* = 1. Abbreviations: ESBL: Extended-spectrum beta-lactamase; MDR: Multidrug-resistant.

**Table 3:** Comparison of baseline and clinical characteristics in 278 episodes of acute graft pyelonephritis with bacteremia in 214 KT recipients according to the multidrug-resistant Gram-negative bacilli etiology.

Variables	Multidrug-Resistant Gram-Negative Bacilli, *n* = 79 (%)	Non-Multidrug-Resistant Gram-Negative Bacilli, *n* = 199 (%)	*p-*Value
Baseline characteristics			
Male sex	64 (81.0)	107 (53.8)	<0.001
Age, years, median (IQR)	69 (61–72)	62 (57–71)	0.023
**Underlying disease**	74 (93.7)	184 (92.5)	0.725
Diabetes mellitus	32 (41.0)	63 (31.7)	0.140
**Prior transplant**	12 (23.5)	34 (25.2)	1.000
**Living donor**	6 (7.6)	19 (9.5)	0.608
**Surgical complications after transplant**	20 (25.3)	23 (11.6)	0.004
Re-intervention	4 (5.1)	9 (3.0)	1.000
Lymphocele	2 (2.5)	2 (1.0)	0.320
Surgical site infection	5 (6.3)	2 (1.0)	0.021
Post-surgical hematoma	3 (3.8)	9 (4.5)	1.000
Ureteral stenosis	3 (3.8)	0	0.022
Other ^1^	2 (2.5)	1 (0.5)	0.195
**TMP-SMZ prophylaxis**	27 (34.2)	55 (27.8)	0.292
**Immunosuppressive therapy**			
Prednisone	72 (91.1)	166 (83.4)	0.098
Median prednisone mg per day (IQR)	5.0 (5.0–10.0)	5.0 (5.0–6.9)	0.325
Anti-calcineurin inhibitors	72 (91.1)	172 (86.4)	0.280
mTOR inhibitors	6 (7.6)	29 (14.6)	0.114
Mycophenolate mofetil	69 (87.3)	179 (89.9)	0.527
Lymphocyte-depleting antibody (≤6 months)	26 (32.9)	62 (31.3)	0.796
Anti-thymocyte globulin (≤6 months)	13 (16.5)	30 (15.2)	0.799
≥1 pulse of 1 g of intravenous methylprednisolone (≤6 months)	7 (9.0)	29 (14.7)	0.203
**Acute allograft rejection** (≤6 months)	6 (7.6)	12 (6.1)	0.640
**Prior antibiotic therapy ^2^**	56 (70.9)	84 (42.2)	<0.001
Prior beta-lactam use	35 (44.3)	62 (31.2)	0.038
Prior carbapenem use	26 (32.9)	15 (7.5)	<0.001
Prior quinolones use	21 (26.6)	23 (11.6)	0.002
Prior glycopeptide use	6 (7.6)	7 (3.5)	0.204
>1 previous antibiotic type ^3^	26 (32.9)	27 (13.6)	<0.001
**Prior bacteremic acute graft pyelonephritis ^4^**	30 (38.0)	34 (17.1)	<0.001
**Median days from kidney transplantation (IQR)**	132 (56–1286)	290 (30–1503)	0.336
**First year after kidney transplantation**	54 (68.4)	106 (53.3)	0.022
**Nosocomial acquisition**	47 (59.5)	76 (38.2)	0.001
**Urinary catheters**			
Use of urethral catheter	28 (35.4)	53 (26.8)	0.152
Use of ureteral catheter	11 (20.8)	33 (25.2)	0.572
Nephrostomy	6 (11.1)	4 (3.1)	0.066
Clinical and laboratory data at admission for acute graft pyelonephritis			
Temperature ≥ 38 °C	40 (51.9)	92 (48.9)	0.656
White blood cell count > 10,000/mm^3^	42 (53.2)	117 (58.8)	0.392
Lymphocyte count < 500/mm^3^	33 (42.9)	88 (44.7)	0.786
Platelet count < 50,000/mm^3^	1 (1.3)	1 (0.5)	0.484
Hypoalbuminemia (<3 g/L)	24 (40.0)	37 (30.8)	0.221

^1^ Other surgical complications after transplantation—MDR Gram-negative bacilli AGP: Renal ischemia = 1, eventration = 1. Non-MDR Gram-negative bacilli AGP: Renal ischemia = 1. ^2^ Prior antibiotic therapy was defined as the receipt of any systemic antibiotic in the preceding month for 48 h or more. ^3^ More than 1 previous antibiotic type—MDR Gram-negative bacilli: 2 antibiotic types, 19; 3 antibiotic types, 7. Non-MDR Gram-negative bacilli: 2 antibiotic types, 24; 3 antibiotic types, 3. ^4^ Prior episode of bacteremic pyelonephritis—MDR Gram-negative bacilli: 49 patients had 1 episode, 21 patients had 2 episodes, 7 patients had 3 episodes, 1 patient had 4 episodes, and 1 patient had 5 episodes. Non-MDR Gram-negative bacilli: 165 patients had 1 episode, 23 patients had 2 episodes, 8 patients had 3 episodes, and 3 patients had 4 episodes. (*p* = 0.001). Abbreviations: IQR, interquartile range; mTOR, mammalian target of rapamycin; TMP–SMZ, trimethoprim–sulfamethoxazole.

**Table 4:** Multivariate analysis of risk factors for MDR Gram-negative bacilli acute graft pyelonephritis with bacteremia in KT recipients.

Variables	OR	95% CI	*p-*Value
Male sex	3.084	1.601–5.939	0.001
Prior bacteremic acute graft pyelonephritis	2.115	1.093–4.093	0.026
Prior antibiotic therapy in the preceding month	2.471	1.335–4.571	0.004
Nosocomial acquisition	2.034	1.142–3.623	0.016

Variables included in the analysis were male sex, age, prior bacteremic acute graft pyelonephritis, prior antibiotic therapy in the preceding month, nosocomial acquisition, surgical complications after kidney transplant, and first year after kidney transplant. Abbreviations: MDR, multidrug-resistant; OR, odds ratio; CI, confidence interval.

**Table 5:** Comparison of outcomes in 278 episodes of acute graft pyelonephritis with bacteremia in 214 KT recipients according to multidrug-resistant Gram-negative bacilli etiology.

Variables	Multidrug-Resistant Gram-Negative Bacilli, *n* = 79 (%)	Non-Multidrug-Resistant Gram-Negative Bacilli, *n* = 199 (%)	*p-*Value
**Complications**	49 (62.0)	110 (55.3)	0.305
Shock at presentation ^1^	5 (6.3)	8 (4.1)	0.530
Renal impairment ^2^	64 (81.0)	155 (77.9)	0.566
Respiratory failure	6 (7.6)	6 (3.0)	0.108
Multi-organ failure	1 (1.3)	1 (0.5)	0.491
Intensive care unit admission	4 (5.1)	8 (4.1)	0.747
Mechanical ventilation	3 (3.8)	5 (2.5)	0.693
**Persistent bacteremia**	6 (7.6)	4 (2.0)	0.034
**Coinfection**	20 (25.3)	19 (9.5)	0.001
**Viral coinfection**	15 (19.5)	14 (7.2)	0.003
Cytomegalovirus	13 (16.5)	8 (4.0)	<0.001
SARS-CoV-2	1	3	
Influenza virus	0	2	
Herpes simplex virus	1	0	
**Fungal coinfection**	3 (3.8)	0 (0)	0.023
Candida species	2	0	
Aspergillus species	1	0	
Inappropriate empirical antibiotic treatment ^3^	34 (43.0)	34 (17.1)	<0.001
Days of intravenous antibiotic (median, IQR)	16 (14–21)	5 (3–7)	<0.001
Days of antibiotic (median, IQR)	21 (14–21)	16 (14–21)	0.093
Days of admission since bacteremia (median, IQR)	16 (10–35)	7 (5–14)	<0.001
Overall 30-day mortality	1 (1.3)	2 (1.0)	1.000

^1^ Septic shock was defined as sepsis-induced hypotension persisting despite adequate fluid resuscitation. ^2^ Renal impairment was defined as a serum creatinine level >1.5 mg/dL. ^3^ Empirical antibiotic therapies were considered inadequate if the treatment regimen did not include at least one antibiotic that was active in vitro against the infecting microorganism. Abbreviations: IQR, interquartile range.

## Data Availability

The data presented in this study are available upon reasonable request from the corresponding author. The data are not publicly available, due to privacy restrictions.

## Supplementary Materials

The following supporting information can be downloaded at https://www.mdpi.com/article/10.3390/jcm11113165/s1: Table S1: Comparison of baseline characteristics of 278 episodes of acute graft pyelonephritis with bacteremia in 214 KT recipients according to gender; Table S2: Comparison of causative microorganisms of 278 episodes of acute graft pyelonephritis with bacteremia in 214 KT recipients according to gender.

## References

[1] Kritikos A., Manuel O. (2016). Bloodstream infections after solid-organ transplantation. Virulence.

[2] Pouch S.M., Patel G., AST Infectious Diseases Community of Practice (2019). Multidrug-resistant Gram-negative bacterial infections in solid organ transplant recipients-Guidelines from the American Society of Transplantation Infectious Diseases Community of Practice. Clin. Transpl..

[3] Cervera C., van Delden C., Gavaldà J., Welte T., Akova M., Carratalà J., ESCMID Study Group for Infections in Compromised Hosts (2014). Multidrug-resistant bacteria in solid organ transplant recipients. Clin. Microbiol. Infect..

[4] Parasuraman R., Julian K., AST Infectious Diseases Community of Practice (2013). Urinary tract infections in solid organ transplantation. Am. J. Transpl..

[5] Vidal E., Torre-Cisneros J., Blanes M., Montejo M., Cervera C., Aguado J.M., Len O., Carratalá J., Cordero E., Bou G. (2012). Bacterial urinary tract infection after solid organ transplantation in the RESITRA cohort. Transpl. Infect. Dis..

[6] Fiorentino M., Pesce F., Schena A., Simone S., Castellano G., Gesualdo L. (2019). Updates on urinary tract infections in kidney transplantation. J. Nephrol..

[7] Kamath N.S., John G.T., Neelakantan N., Kirubakaran M.G., Jacob C.K. (2006). Acute graft pyelonephritis following renal transplantation. Transpl. Infect. Dis..

[8] Giral M., Pascuariello G., Karam G., Hourmant M., Cantarovich D., Dantal J., Blancho G., Coupel S., Josien R., Daguin P. (2002). Acute graft pyelonephritis and long-term kidney allograft outcome. Kidney Int..

[9] Maanaoui M., Baes D., Hamroun A., Khedjat K., Vuotto F., Faure E., Lopez B., Bouyé S., Caes T., Lionet A. (2021). Association between acute graft pyelonephritis and kidney graft survival: A single-center observational study. Am. J. Transpl..

[10] Linares L., García-Goez J.F., Cervera C., Almela M., Sanclemente G., Cofán F., Ricart M.J., Navasa M., Moreno A. (2009). Early bacteremia after solid organ transplantation. Transpl. Proc..

[11] Pellé G., Vimont S., Levy P.P., Hertig A., Ouali N., Chassin C., Arlet G., Rondeau E., Vandewalle A. (2007). Acute pyelonephritis represents a risk factor impairing long-term kidney graft function. Am. J. Transpl..

[12] Lorenz E.C., Cosio F.G. (2010). The impact of urinary tract infections in renal transplant recipients. Kidney Int..

[13] Fiorante S., Fernández-Ruiz M., López-Medrano F., Lizasoain M., Lalueza A., Morales J.M., San-Juan R., Andrés A., Otero J.R., Aguado J.M. (2011). Acute graft pyelonephritis in renal transplant recipients: Incidence, risk factors and long-term outcome. Nephrol. Dial. Transpl..

[14] Sabé N., Cruzado J.M., Carratalà J. (2021). Asymptomatic bacteriuria in kidney transplant recipients: To treat or not to treat-that is the question. Clin. Microbiol. Infect..

[15] Origüen J., Fernández-Ruiz M., López-Medrano F., Ruiz-Merlo T., González E., Morales J.M., Fiorante S., San-Juan R., Villa J., Orellana M.Á. (2016). Progressive increase of resistance in Enterobacteriaceae urinary isolates from kidney transplant recipients over the past decade: Narrowing of the therapeutic options. Transpl. Infect. Dis..

[16] Oriol I., Sabé N., Simonetti A.F., Lladó L., Manonelles A., González J., Tubau F., Carratalà J. (2017). Changing trends in the aetiology, treatment and outcomes of bloodstream infection occurring in the first year after solid organ transplantation: A single-centre prospective cohort study. Transpl. Int..

[17] Bodro M., Sanclemente G., Lipperheide I., Allali M., Marco F., Bosch J., Cofan F., Ricart M.J., Esforzado N., Oppenheimer F. (2015). Impact of antibiotic resistance on the development of recurrent and relapsing symptomatic urinary tract infection in kidney recipients. Am. J. Transpl..

[18] Magiorakos A.P., Srinivasan A., Carey R.B., Carmeli Y., Falagas M.E., Giske C.G., Harbarth S., Hindler J.F., Kahlmeter G., Olsson-Liljequist B. (2012). Multidrug-resistant, extensively drug-resistant and pandrug-resistant bacteria: An international expert proposal for interim standard definitions for acquired resistance. Clin. Microbiol. Infect..

[19] Evans L., Rhodes A., Alhazzani W., Antonelli M., Coopersmith C.M., French C., Machado F.R., Mcintyre L., Ostermann M., Prescott H.C. (2021). Executive Sumary: Surviving Sepsis Campaign: International Guidelines for the Management of Sepsis and Septic Shock 2021. Crit. Care Med..

[20] Tsikala-Vafea M., Basoulis D., Pavlopoulou I., Darema M., Deliolanis J., Daikos G.L., Boletis J., Psichogiou M. (2020). Bloodstream infections by gram-negative bacteria in kidney transplant patients: Incidence, risk factors, and outcome. Transpl. Infect. Dis..

[21] Berenger B.M., Doucette K., Smith S.W. (2016). Epidemiology and risk factors for nosocomial bloodstream infections in solid organ transplants over a 10-year period. Transpl. Infect. Dis..

[22] Gomila A., Carratalà J., Eliakim-Raz N., Shaw S., Tebé C., Wolkewitz M., Wiegand I., Grier S., Vank C., Cuperus N. (2019). Clinical outcomes of hospitalised patients with catheter-associated urinary tract infection in countries with a high rate of multidrug-resistance: The COMBACTE-MAGNET RESCUING study. Antimicrob. Resist. Infect. Control.

[23] De Oliveira Marinho A.C., Tavares-da-Silva E., Bastos C.A., Roseiro A., Parada B., Retroz E., Marconi L., Moreira P., Nunes P., Simões P. (2021). Acute Urinary Retention After Kidney Transplant: Effect on Graft Function, Predictive Factors, and Treatment. Transpl. Proc..

[24] Hurst F.P., Neff R.T., Falta E.M., Jindal R.M., Lentine K.L., Swanson J.S., Agodoa L.Y., Abbott K.C. (2009). Incidence, predictors, and associated outcomes of prostatism after kidney transplantation. Clin. J. Am. Soc. Nephrol..

[25] Yuan X., Liu T., Wu D., Wan Q. (2018). Epidemiology, susceptibility, and risk factors for acquisition of MDR/XDR Gram-negative bacteria among kidney transplant recipients with urinary tract infections. Infect. Drug Resist..

[26] Wu X., Dong Y., Liu Y., Li Y., Sun Y., Wang J., Wang S. (2016). The prevalence and predictive factors of urinary tract infection in patients undergoing renal transplantation: A meta-analysis. Am. J. Infect. Control.

[27] Bodro M., Sabé N., Tubau F., Lladó L., Baliellas C., Roca J., Cruzado J.M., Carratalà J. (2013). Risk factors and outcomes of bacteremia caused by drug-resistant ESKAPE pathogens in solid-organ transplant recipients. Transplantation.

[28] Anesi J.A., Lautenbach E., Tamma P.D., Thom K.A., Blumberg E.A., Alby K., Bilker W.B., Werzen A., Tolomeo P., Omorogbe J. (2021). Risk Factors for Extended-Spectrum beta-lactamase-Producing Enterobacterales Bloodstream Infection Among Solid-Organ Transplant Recipients. Clin. Infect. Dis..

[29] Pinheiro H.S., Mituiassu A.M., Carminatti M., Braga A.M., Bastos M.G. (2010). Urinary tract infection caused by extended-spectrum beta-lactamase-producing bacteria in kidney transplant patients. Transpl. Proc..

[30] Bodro M., Sabé N., Tubau F., Lladó L., Baliellas C., González-Costello J., Cruzado J.M., Carratalà J. (2015). Extensively drug-resistant Pseudomonas aeruginosa bacteremia in solid organ transplant recipients. Transplantation.

[31] Kalil A.C., Syed A., Rupp M.E., Chambers H., Vargas L., Maskin A., Miles C.D., Langnas A., Florescu D.F. (2015). Is bacteremic sepsis associated with higher mortality in transplant recipients than in nontransplant patients? A matched case-control propensity-adjusted study. Clin. Infect. Dis..

[32] Oriol I., Sabé N., Melilli E., Lladó L., González-Costello J., Soldevila L., Carratalà J. (2015). Factors influencing mortality in solid organ transplant recipients with bloodstream infection. Clin. Microbiol. Infect..

[33] Hamandi B., Holbrook A.M., Humar A., Brunton J., Papadimitropoulos E.A., Wong G.G., Thabane L. (2009). Delay of adequate empiric antibiotic therapy is associated with increased mortality among solid-organ transplant patients. Am. J. Transpl..

[34] Lizza B.D., Betthauser K.D., Ritchie D.J., Micek S.T., Kollef M.H. (2021). New Perspectives on Antimicrobial Agents: Ceftolozane-Tazobactam. Antimicrob. Agents Chemother..

[35] McCreary E.K., Heil E.L., Tamma P.D. (2021). New Perspectives on Antimicrobial Agents: Cefiderocol. Antimicrob. Agents Chemother..

[36] Zhang F., Zhong J., Ding H., Liao G. (2021). Efficacy of Ceftazidime-Avibactam in the Treatment of Carbapenem-Resistant Klebsiella pneumoniae Infection After Kidney Transplantation. Infect. Drug Resist..

