# Supplementary material for: Risk Factors and Outcomes of Acute Graft Pyelonephritis with Bacteremia Due to Multidrug-Resistant Gram-Negative Bacilli among Kidney Transplant Recipients
Source: J Clin Med. 2022 Jun 2;11(11):3165. doi: 10.3390/jcm11113165 (PMC9181603; doi:10.3390/jcm11113165)
Supplement: Supplementary file 1 [file jcm-11-03165-s001.zip › jcm-1726321-supplementary.pdf]

Supplementary Table S1. Comparison of baseline characteristics of 278 episodes of acute graft pyelonephritis with bacteremia in 214 KT recipients according to gender.

Variables	Male Kidney Transplant Recipients *n* = 171 (%)	Female Kidney Transplant Recipients *n*= 107 (%)	*p* Value
**Age, years, median (IQR)**	67 (59–71)	65 (53–71)	0.065
**Underlying disease**	159 (93.0)	99 (92.5)	0.885
Diabetes mellitus	59 (34.7)	36 (33.6)	0.856
**Prior transplant**	24 (22.2)	22 (28.2)	0.351
**Living donor**	17 (9.9)	8 (7.5)	0.485
**Surgical complications after transplant**	28 (164.4)	15 (14.0)	0.597
**TMP-SMZ prophylaxis**	54 (31.8)	28 (26.2)	0.320
**Immunosuppressive therapy**			
Prednisone	152 (88.9)	86 (80.4)	0.049
Anticalcineurin inhibitors	145 (84.8)	99 (92.5)	0.061
mTOR inhibitors	25 (14.6)	10 (9.3)	0.197
Mycofenolate mofetil	151 (88.3)	97 (90.7)	0.539
Lymphocyte-depleting antibody (≤6 months)	59 (34.7)	29 (27.1)	0.186
Antithymocyte globulin (≤6 months)	29 (17.1)	14 (13.2)	0.391
≥1 pulse of 1 g of intravenous methylprednisolone (≤6 months)	26 (15.3)	10 (9.5)	0.168
**Acute allograft rejection** (≤6 months)	10 (5.8)	8 (7.5)	0.577
**Prior antibiotic therapy** [1]	92 (53.8)	48 (44.9)	0.147
Prior beta-lactam use	61 (35.7)	36 (33.6)	0.796
Prior carbapenem use	32 (18.7)	9 (8.4)	0.018
Prior quinolone use	24 (14.0)	20 (18.7)	0.301
Prior glycopeptide use	13 (7.6)	0	0.003
**Prior episode of bacteremic acute graft pyelonephritis**	45 (26.3)	19 (17.8)	0.099
**Median days from kidney transplant (IQR)**	199 (59–71)	281 (60–1161)	0.512
**First year after kidney transplant**	103 (60.2)	57 (53.3)	0.253
**Nosocomial acquisition**	85 (49.7)	38 (35.5)	0.020
**Urinary catheters**			
Use of urethral catheter	61 (35.7)	20 (18.9)	0.003
Use of ureteral catheter	30 (27.3)	14 (18.9)	0.193
Nephrostomy	8 (7.2)	2 (2.7)	0.320

[1] Prior antibiotic therapy was defined as the receipt of any systemic antibiotic in the preceding month for 48 h or more. Abbreviations: IQR, interquartile range; mTOR, mammalian target of rapamycin; TMP–SMZ, trimethoprim–sulfamethoxazole.

Supplementary Table S2. Comparison of causative microorganisms of 278 episodes of acute graft pyelonephritis with bacteremiain 214 KT recipients according to gender.

Microorganisms	Male Kidney Transplant Recipients *n* = 171 (%)	Female Kidney Transplant Recipients *n* = 107 (%)	*p* Value
**Gram-negative**			
Escherichia coli	59 (34.5)	71 (66.4)	<0.001
ESBL-producing *Escherichia coli*	12 (7.0)	8 (7.5)	0.885
Klebsiella spp	47 (27.5)	25 (23.4)	0.445
ESBL-producing *Klebsiella* spp	35 (20.5)	6 (5.6)	0.001
Carbapenemase-producing*Klebsiella* spp	4 (2.3)	0	0.302
Pseudomonas spp	32 (18.7)	3 (2.8)	<0.001
MDR Pseudomonas	16 (9.4)	1 (0.9)	0.004
Enterobacter spp	7 (4.1)	2 (1.9)	0.490
Proteus mirabillis	6 (3.5)	1 (0.9)	0.256
Acinetobacter baumannii	1 (0.6)	0	1.000
Multi drug resistant Gram-negative bacilli	64 (37.4)	15 (14.0)	<0.001
**Gram-positive**			
Enterococcus spp	16 (9.4)	2 (1.9)	0.014
Enterococcus faecium	3 (7.6)	1 (0.9)	0.013
Staphylococcus aureus	3 (1.8)	1 (0.9)	1.000

Abbreviations: ESBL: Extended-spectrum beta-lactamases; MDR: Multi-drug resistant.
